# Temporal trends in tolvaptan use after revision of national heart failure guidelines in Japan

**DOI:** 10.1038/s41598-021-98173-8

**Published:** 2021-09-29

**Authors:** Yusuke Yamazaki, Yasuyuki Shiraishi, Shun Kohsaka, Yuji Nagatomo, Keiichi Fukuda, Takashi Kohno, Tsutomu Yoshikawa

**Affiliations:** 1 Department of Cardiology, Keio University School of Medicine, 35 Shinanomachi Shinjuku-ku, Tokyo, 160-8582 Japan; 2 Department of Cardiology, National Defence Medical College Hospital, Tokorozawa, Japan; 3 Department of Cardiovascular Medicine, Kyorin University Faculty of Medicine, Tokyo, Japan; 4 Department of Cardiology, Sakakibara Heart Institute, Tokyo, Japan

**Keywords:** Cardiology, Medical research

## Abstract

Within no definite diuretic protocol for acute heart failure (AHF) patients and its variation in regional clinical guidelines, the latest national guidelines in Japan commends use of tolvaptan in diuretic-resistant patients. This study aimed to examine trends in tolvaptan usage and associated outcomes of AHF patients requiring hospitalization. Between April, 2018 and October, 2019, 1343 consecutive AHF patients (median 78 [69–85] year-old) were enrolled in a prospective, multicenter registry in Japan. Trends over time in tolvaptan usage, along with the severity of heart failure status based on the Get With The Guideline-Heart Failure [GWTG-HF] risk score, and in-hospital outcomes were investigated. During the study period, tolvaptan usage has increased from 13.0 to 28.7% over time (*p* for trend = 0.07), and 49.4% started tolvaptan within 3 days after admission. The GWTG-HF risk score in the tolvaptan group has significantly decreased over time, while that in the non-tolvaptan group has unchanged. There were no differences in the in-hospital mortality rate between the patients with and without tolvaptan (6.7% vs. 5.8%). After revision of the Japanese clinical practice guidelines for AHF in March 2018, tolvaptan usage for AHF patients has steadily increased. However, in-hospital outcomes including mortality do not seem to be affected.

## Introduction

Clinical congestion is the primary pathophysiology that leads to impaired exercise capacity and heart failure (HF) hospitalization. Diuretics continue to play an essential role in the treatment to relieve congestive signs and symptoms. Meanwhile, natriuretic agents (i.e., loop and/or thiazide-type diuretics) are also known to have a negative impact on clinical outcomes in patients with HF^1,2^. In this context, clinical practice guidelines generally recommend use of natriuretic agents to achieve and maintain euvolemia with the lowest achievable dose^3,4^.

Tolvaptan, a vasopressin-2 receptor antagonist, leads to aquaresis without natriuresis-related electrolyte disturbances and is widely implemented in the modern HF management in Japan^5^. Originally, the Japanese Circulation Society (JCS) and Japanese Heart Failure Society (JHFS) 2017 guideline on Diagnosis and Treatment of Acute and Chronic Heart Failure commend use of tolvaptan with low dose (7.5–15 mg/day) for the treatment of fluid retention in patients not responding well to other diuretics including loop diuretics (Class IIa, Level of Evidence A)^6^. Favorable results on the surrogate outcomes (e.g., 48-h urine volume) were demonstrated in the domestic trials with the usage of low-dose tolvaptan in Japan^7,8^, albeit more large-scale trials that were performed in Western countries failed to show the effect of a high-dose tolvaptan (30 mg/day) on improving the prognosis as well as HF symptoms in patients with acute heart failure (AHF)^9–11^.

These recommendations may have led to use of tolvaptan in a broader range of AHF patients. Herein, we aimed to examine the usage of tolvaptan by their risk profile in the Japanese inpatient-based multicenter registry of AHF patients. We also assessed an association of tolvaptan usage with in-hospital outcomes after revision of the JCS/JHFS clinical practice guidelines.

## Results

### Patient characteristics

**Figure 1:**
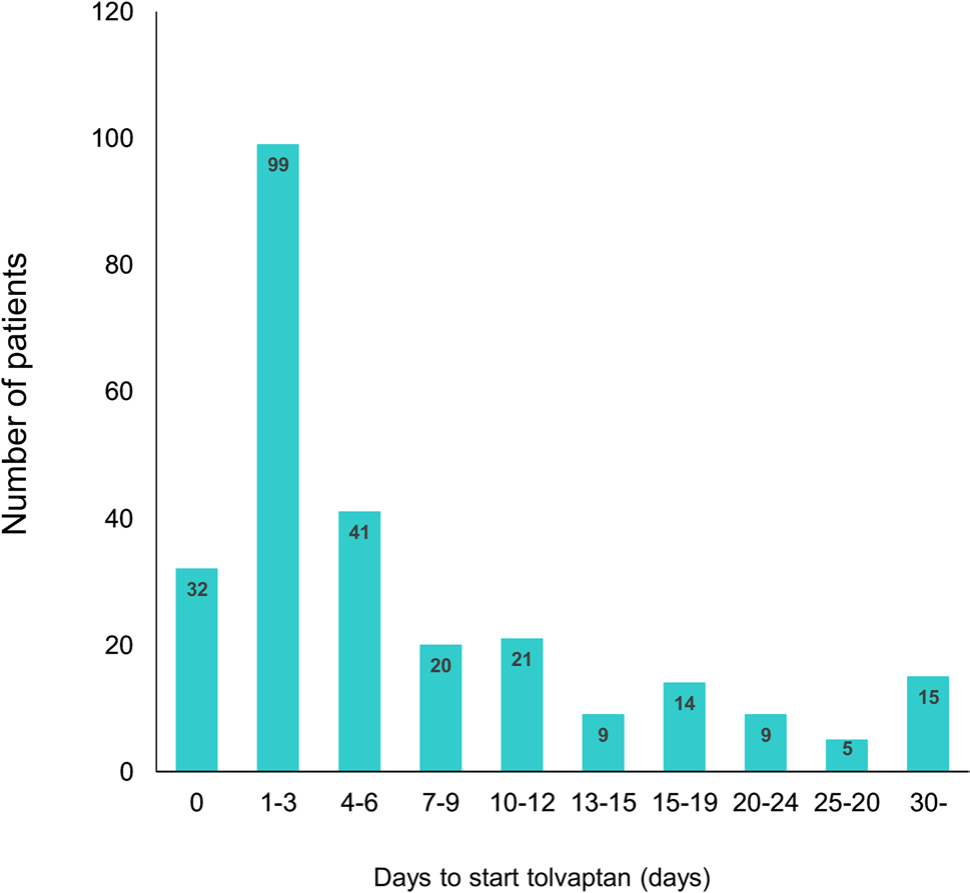
Timing of tolvaptan usage during the index hospitalization. The median time of initiation of tolvaptan from hospitalization was 3 days.

In a total of 1343 patients, the mean age of the patients was 78 (interquartile range [IQR] 69–85) years, and 62% were men with a systolic blood pressure, heart rate, and left ventricular ejection fraction of 135 (IQR 116–160) mmHg, 92 (IQR 74–112) beat per minute, and 45% (IQR 31–57%), respectively. Overall, 267 (19.9%) patients received oral tolvaptan during their index hospitalization. A half (n = 132) of the tolvaptan group (an approved dose of tolvaptan 7.5–15 mg/day in Japan) started an add-on tolvaptan to loop diuretics within 3 days after admission (Fig. 1).

Baseline characteristics of the AHF patients at the time of admission were compared by dividing into two groups based on use of tolvaptan during their hospitalization (Table 1). Compared with the non-tolvaptan group, the tolvaptan group had a higher serum creatinine level (1.21 [IQR 0.88–1.72] mg/dL vs.1.07 [IQR 0.82–1.46] mg/dL; p < 0.001) and a lower hemoglobin level (11.6 [IQR 9.8–13.2] g/dL vs. 12.3 [IQR 10.6–13.8] g/dL; p < 0.001). As for medication before admission, the tolvaptan group tended to frequently use loop diuretics (61% vs. 44%; p < 0.001) and mineralocorticoid receptor antagonists (27% vs. 17%; p < 0.001). Also, the patient characteristics at the time of discharge by the tolvaptan and non-tolvaptan groups were presented in Table S1 in the “Online Supplement”.

**Table 1:** Baseline characteristics.

	Tolvaptan (−) n = 1076	Tolvaptan (+) n = 267	p value
Age (years)	79 (68–86)	79 (71–85)	0.38
Men, n (%)	678 (64)	161 (60)	0.41
Body mass index (kg/m^2^)	22.7 (20.2–26.1)	23.4 (20.5–26.0)	0.27
Systolic BP (mmHg)	136 (117–161)	134 (115–157)	0.17
Heart rate (bpm)	94 (75–114)	87 (73–105)	0.11
Left ventricular ejection fraction (%)	44 (32–57)	45 (31–58)	0.59
GWTG-HF risk score	41 (35–47)	42 (36–48)	0.05
**Etiology, n (%)**			0.03
DCM	152 (14)	22 (8)	
ICM	256 (24)	66 (25)	
Valvular	233 (22)	73 (27)	
Others	435 (40)	106 (40)	
**Comorbidities, n (%)**			
History of HF hospitalization	381 (35)	102 (38)	0.40
Coronary artery disease	288 (27)	80 (30)	0.35
Atrial fibrillation	449 (41)	118 (45)	0.47
Hypertension	651 (62)	166 (64)	0.84
Diabetes mellitus	334 (31)	89 (34)	0.47
Dyslipidemia	352 (33)	103 (39)	0.07
Stroke	128 (12)	41 (14)	0.13
COPD	56 (5)	9 (3)	0.27
Dementia	103 (10)	13 (5)	0.02
**Laboratory findings**			
Hemoglobin (g/dL)	12.3 (10.6–13.8)	11.6 (9.8–13.2)	< 0.001
Creatinine (mg/dL)	1.07 (0.82–1.46)	1.21 (0.88–1.72)	< 0.001
BUN (mg/dL)	22.8 (17.0–34.4)	26.3 (18.6–41.1)	< 0.001
Sodium (mEq/L)	140.0 (138.0–142.0)	139.0 (136.0–141.0)	< 0.001
Albumin (mg/dL)	3.5 (3.2–3.8)	3.5 (3.2–3.9)	0.71
BNP (pg/mL)^a^	763 (444–1322)	822 (499–1414)	0.15
NT-proBNP (pg/mL)^a^	4235 (2457–7860)	4846 (2380–9678)	0.16
**Medication before admission, n (%)**			
ACEI or ARB	402 (38)	105 (43)	0.55
Beta blocker	467 (42)	132 (49)	0.08
MRA	185 (17)	73 (27)	< 0.001
Digoxin	33 (3)	10 (4)	0.56
Loop diuretics	479 (44)	162 (61)	< 0.001
Furosemide equivalent (mg)^b^	0 (0–20)	20 (0–40)	0.29
Thiazide-type diuretics	37 (4)	12 (4)	0.47
**In-hospital treatment, n (%)**			
Loop diuretics, iv	871 (81)	225 (84)	0.21
Vasodilators, iv	359 (33)	93 (35)	0.65
Inotropes, iv	118 (11)	35 (13)	0.32
Non-invasive ventilation	386 (36)	126 (47)	< 0.001
Intubation	34 (3)	11 (4)	0.45
IABP	19 (2)	6 (2)	0.61
VA-ECMO/VAD	9 (< 1)	1 (< 1)	0.70

*BP* blood pressure, *DCM* dilated cardiomyopathy, *ICM* ischemic cardiomyopathy, *HF* heart failure, *COPD* chronic obstructive pulmonary disease, *eGFR* estimated glomerular filtration rate, *BUN* blood urea nitrogen, *BNP* B-type natriuretic peptide, *NT-proBNP* N-terminal pro-B-type natriuretic peptide, *ACEI* angiotensin-converting enzyme inhibitor, *ARB* angiotensin receptor blocker, *MRA* mineralocorticoid receptor antagonist, *IABP* intraaortic balloon pumping, *VA-ECMO* veno-arterial extracorporeal membrane oxygenation, *VAD* ventricular assist device.

^a^In the 959 patients, BNP levels were measured, in contrast, NT-proBNP levels were measured in 384 patients.

^b^Furosemide 20 mg = Azosemide 30 mg = Torsemide 4 mg.

### Time trend of tolvaptan usage and GWTG-HF risk score

**Figure 2:**
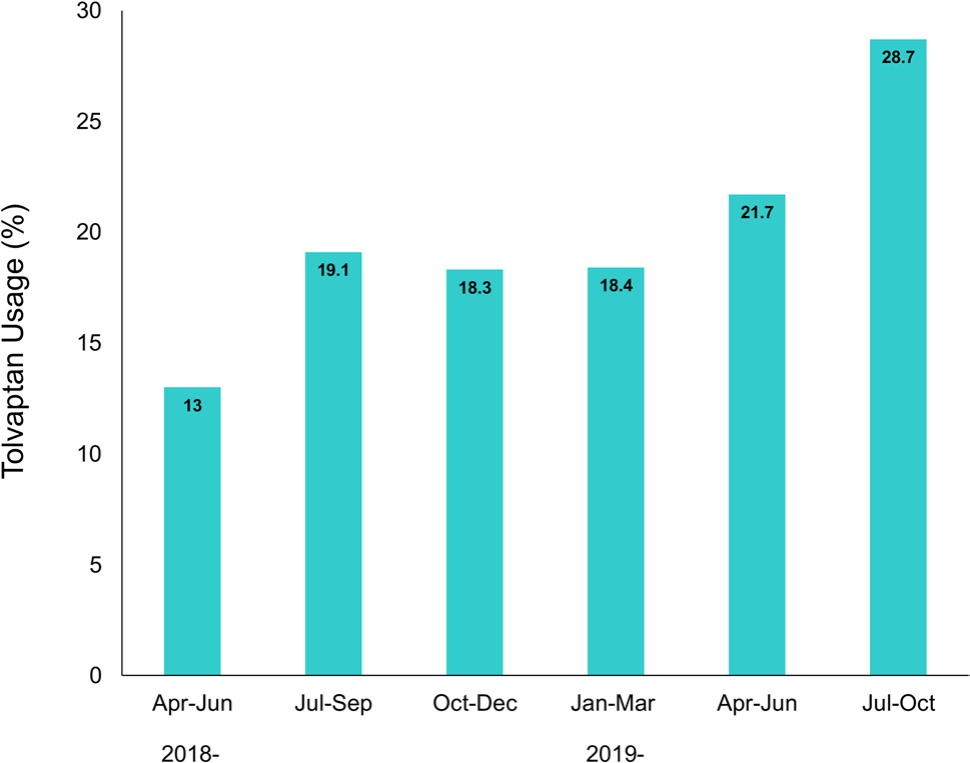
Temporal trend for tolvaptan usage after revision of the JCS/JHFS guidelines. The usage of tolvaptan in the acute setting increased steadily but no statistical significance (*p* for trend = 0.07).

**Figure 3:**
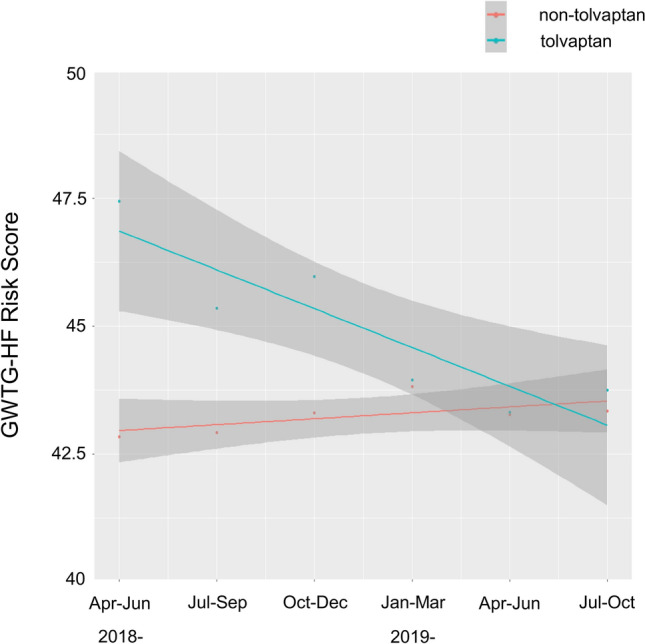
Temporal trends for disease severity by the Get With The Guideline-Heart Failure [GWTG-HF] risk score. The GWTG-HF risk score in the tolvaptan group significantly decreased from 47 to 41 (*p* for trend = 0.015), while that in the non-tolvaptan group remained unchanged.

During the study period after revision of the JCS/JHFS clinical practice guidelines on March 2018, the tolvaptan usage steadily increased from 13.0 to 28.7% over time, but with no statistical significance (*p* for trend = 0.07) (Fig. 2). Notably, the average Get With The Guideline-Heart Failure (GWTG-HF) risk score in the tolvaptan group has significantly decreased from 47 to 41 over the study period (*p* for trend = 0.015), while the score in the non-tolvaptan group remained unchanged (Fig. 3).

### Tolvaptan usage and in-hospital outcomes

Regardless of tolvaptan usage, no significant difference in the in-hospital mortality rate was observed (6.7% in the tolvaptan group vs. 5.8% in the non-tolvaptan group, p = 0.56), even after adjusted for the GWTG-HF risk score. Meanwhile, the tolvaptan group had a longer length of hospital stay (LOHS) compared with the non-tolvaptan group (23 [IQR 14–33] days vs. 16 [IQR 9–24] days; p < 0.001).

Table 2 shows the change in clinical and laboratory data of the patients who were successfully discharged. In the tolvaptan group, body weight significantly decreased by 5.4 kg [IQR 3.0–8.5] compared with that in the non-tolvaptan group (4.4 kg [IQR 2.2–7.3]; p = 0.004). Meanwhile, the improvement rate of natriuretic peptide (NP) levels from admission to discharge in the tolvaptan group was significantly lower than that in the non-tolvaptan group: 56.9% [IQR 25.3–74.9] in the tolvaptan group vs. 62.3% [IQR 38.2–80.4] in the non-tolvaptan group (p = 0.006). Even in the multivariable linear regression model adjusting for the GWTG-HF risk score, tolvaptan usage remained to be associated with a lower improvement rate of NP levels (p = 0.006). In addition, there was no difference in a change in serum creatinine levels from admission to discharge between the two groups (0.05 mg/dL [95% confidence interval − 0.04 to 0.15] in the tolvaptan group vs. − 0.02 mg/dL [95% confidence interval − 0.07 to 0.03] in the non-tolvaptan group, p = 0.16 for ANCOVA).

**Table 2:** Changes in clinical variables during the index hospitalization.

	Tolvaptan (−) n = 1014	Tolvaptan (+) n = 249	p value
Body weight (kg)	− 4.4 (− 7.3 to − 2.2)	− 5.4 (− 8.5 to − 3.0)	0.004
Systolic BP (mmHg)	− 25 (− 47 to − 7.0)	− 25 (− 45 to − 6)	0.58
Heart rate (bpm)	− 20 (− 42 to − 3.0)	− 13 (− 30 to 0)	< 0.001
Hemoglobin (g/dL)	0 (− 0.9 to 0.9)	− 0.1 (− 1.2 to 1.0)	0.16
Creatinine (mg/dL)	− 0.02 (− 0.07 to 0.03)	0.05 (− 0.04 to 0.15)	0.16
BUN (mg/dL)	0.40 (− 6.1 to 6.3)	2.0 (− 5.0 to 9.9)	0.005
Sodium (mEq/L)	− 1.0 (− 3.0 to 1.7)	0 (− 3.0 to 3.0)	0.035
Albumin (mg/dL)	− 0.10 (− 0.40 to 0.20)	− 0.10 (− 0.50 to 0.20)	0.16
Natriuretic peptides (%)^a^	62.3 (38.3–80.4)	56.9 (25.3–74.9)	0.006

^a^The change in natriuretic peptide (NP) levels, including either B-type natriuretic peptide or N-terminal pro-B-type natriuretic peptide, was defined as ([NP values at admission − NP values at discharge]/NP values at admission).

When dividing the patients receiving tolvaptan into two groups by the median time of initiation of tolvaptan, the early tolvaptan group that started tolvaptan within 3 days from hospitalization, compared with the late tolvaptan group (four or more days after hospitalization), showed almost similar baseline characteristics, other than serum creatinine levels: 1.39 [IQR 0.96–1.93] mg/dL in the early group vs. 1.03 [IQR 0.83–1.62] mg/dL in the late group (p < 0.001) (Table S2 in the “Online Supplement”). No statistical difference in the GWTG-HF risk score was observed between the early and late tolvaptan groups. The early group has a shorter LOHS than the late group (20 [IQR 12–30] days vs. 28 [IQR 19–43] days; p < 0.001), while no difference in the in-hospital mortality rate was observed between the two groups (6.8% in the early group vs. 6.6% in the late group; p = 0.58).

## Discussions

Using this prospective multicenter registry (West Tokyo Heart Failure Registry 2 [WET-HF2]), we demonstrated the following findings: (1) tolvaptan tends to be significantly used in patients with renal impairment as well as those who had loop diuretics and mineralocorticoid receptor antagonists at baseline; (2) during the study period, tolvaptan usage has increased from 13.0 to 28.7% over time, but with no statistical significance; (3) in the tolvaptan group, the average GWTG-HF risk score has significantly decreased albeit that in the non-tolvaptan group has unchanged over the study period; and (4) irrespective of tolvaptan usage, there was no between-difference in the in-hospital mortality rate.

Within the international clinical practice guidelines and expert's statements of the treatment for AHF patients, up-titration of intravenous loop diuretics is emphasized preferentially, while use of other diuretics in combination is commended after administration of a substantial dose of loop diuretics to reach the ceiling dose^3,4^. The JCS/JHFS 2017 guidelines also state a directionally similar approach to diuretic-resistant cases^6^. In this context, the nationwide claim-based database in Japan (n = 235,487) reported that tolvaptan usage for AHF patients increased steadily from 3.2% in 2011 to 39.0% in 2018 and the timing of tolvaptan usage became gradually early, and consequently tolvaptan was used within 2 days after admission among more than half of patients who received tolvaptan^5^, which is comparable with our data. On another front, our data as with other observational studies showed that the baseline diuretic dose (20–40 mg furosemide equivalent) in Japanese patients was historically lower than that in Western patients^12,13^. Intravenous administration of loop diuretics for AHF patients is also quantitatively low in Japanese clinical practice: a multicenter observational study described that the median dose of intravenous furosemide was 60 mg (IQR 20–100 mg) within 48 h of admission in AHF patients^14^. Despite a lack of clear reasons on low-dose diuretics in Japan, it may be explained by differences in pharmacokinetics and/or body weight between ethnicities. Although we have no data on the dose of intravenous furosemide and are unable to examine an association of up-titration of loop diuretics and add-on tolvaptan with patient outcomes, results from our study and the previous study^5^ seem to show that physicians in Japan preferentially implement the initial sequential nephron blockade strategy with tolvaptan relative to up-titration of loop diuretics in clinical practice. In the United States, however, one fifth to one third of inpatients with HF had in-hospital treatment escalated beyond initial intravenous diuretic therapy (i.e., up-titration of intravenous diuretics)^15^, while 30–40% of AHF patients in Japan added tolvaptan into intravenous furosemide, reflecting a possibility that the initial dose of loop diuretics for AHF patients may not be sufficient in Japan. Future research is warranted to revalidate the position of 2nd-line diuretics, such as tolvaptan and thiazide-type diuretics, in the treatment for HF and identify patients who can benefit from tolvaptan, given that no clear recommendation with robust clinical evidence to the best diuretic regimen for diuretic-resistant cases.

This study also found no association of tolvaptan usage with in-hospital mortality, regardless of the severity of HF status based on the GWTG-HF risk score and the timing of its administration, as with the results from prior clinical trials^7,9–11^. The GWTG-HF risk score was slightly higher in the tolvaptan group than the non-tolvaptan group (42 points vs. 41 points) and was independently associated with the in-hospital mortality in our study, although the impact of the 1-point increment of the GWTG-HF risk score on the mortality seems modest. On the contrary, the tolvaptan group had a longer LOHS with a greater reduction in body weight and a lower improvement rate of NP levels during hospitalization. This can be in part explained by more patients at advanced stages of HF in the tolvaptan group, reflecting renal impairment, anemia, and higher GWTG-HF risk scores. Short-term administration of tolvaptan leading to aquaresis may be also associated with a blunt responsiveness of NP levels. Given the pharmacological mechanism, tolvaptan removes both intravascular and extravascular volumes equally, and thus NP levels that largely reflect elevated intracardiac filling pressures due to excess of the intravascular volume could be lower in patients with tolvaptan than those who were mainly treated with natriuretic agents. Accumulating evidence suggests that the major component of volume loss with natriuretic agents is derived from the extravascular compartment^16^. Therefore, the time needed for distribution of fluid out of extravascular spaces is a key factor to achieve balanced decongestive therapies. To discriminate unbalanced congestive status, several biomarkers (i.e., carbohydrate antigen 125, soluble CD146, adrenomedullin, and etc.) are known to be independently linked with excessive volume overload beyond NP levels^17^. Further investigation on these reliable biomarkers for clinical congestion and differences in their responsiveness to specific treatments is warranted in the HF patient population.

## Limitation

For a thorough understanding of our results, several limitations should be acknowledged. First, this study was not based on data from randomized controlled trial, and thus unknown confounders may have influenced the results. Second, our findings may not be applicable to other countries as well as other regions of Japan because the WET-HF2 registry consists of eight tertiary hospitals around the Tokyo area though the patient backgrounds in the WET-HF2 registry are comparable with other nationwide registries in Japan^18^. Third, in this study we did not assess long-term outcomes, such as post-discharge mortality, rehospitalization, and also quality of life in AHF patients. A clinically substantial improvement of NP levels during the index hospitalization was seen in both the tolvaptan and non-tolvaptan groups, though its impact on post-discharge outcomes may differ by each treatment. However, the Efficacy of Vasopressin Antagonism in Heart Failure Outcome Study With Tolvaptan (EVEREST), a large-scale, randomized, double-blind, placebo-controlled trial has shown no clinical benefits from use of tolvaptan, including cardiovascular death or HF hospitalization, in patients hospitalized for AHF^19^. We performed an ancillary analysis assessing the association of tolvaptan usage with long-term outcomes, including all-cause death and HF rehospitalization, using our dataset from a previous registration system (WET-HF1 from 2006 to 2017; n = 4000). The patient background of the WET-HF1 and our present registration system (WET-HF2) remain largely comparable^20^. Within the WET-HF1 registry, 168 (4.2%) patients had oral tolvaptan after discharge and during the median follow-up period of 2.0 years, there was no difference in the composite of all-cause death or HF rehospitalization between patients receiving or not receiving tolvaptan after discharge even after the multivariable adjustment and propensity score matching analysis. Finally, we did assess renal function with serum creatinine levels at two time points (admission and discharge), but we do not have data on their highest levels during the index hospitalization, which can misjudge the effect of tolvaptan on the serum creatinine level.

## Conclusion

After revised the clinical practice guidelines for HF in Japan, tolvaptan usage for AHF patients has increased steadily, and tended to be used in lower-risk patients. However, in-hospital outcomes including mortality do not seem to be affected. It may be necessary to reconsider the role of tolvaptan, and further investigation on long-term outcomes is needed to identify patients who can benefit from tolvaptan, particularly given the relative high-cost associated with use of tolvaptan.

## Methods

### Study design and participants

The West Tokyo Heart Failure Registry (WET-HF) was launched in 2006 and thereafter added several institutions to several facilities. From January, 2006 to December, 2017, patients hospitalized for AHF were registered at 6 tertiary hospitals (Keio University Hospital, Kyorin University Hospital, Sakakibara Heart Institute, St. Luke’s International Hospital, Saitama Medical University International Medical Center, and Saiseikai Central Hospital) in the Tokyo area^21^. After newly added 2 institutions (National Defence Medical College Hospital and National Hospital Organization Tokyo Medical Center) into the facilities, the WET-HF2 registry reinitiated since April, 2018 with an update of the collecting variables, including use of tolvaptan during the index hospitalization. The WET-HF2 is a prospective multicenter cohort registry designed to collect data on clinical backgrounds and outcomes from consecutive HF patients who were hospitalized for requiring urgent treatment within 8 tertiary care hospitals in Tokyo, Japan. Individual cardiologists made the clinical diagnosis of AHF at each institution based on the Framingham criteria^22^ and the level of NPs: B-type natriuretic peptide ≥ 100 pg/mL or N-terminal pro-B-type natriuretic peptide ≥ 300 pg/mL at the time of hospitalization. Specifically, patients presenting with acute coronary syndrome or those with < 20 years old were not included in this database.

To assess the care and patient outcomes, baseline data and outcomes were collected by trained clinical research coordinators using a web-based electronic data capture system. To ensure consecutive case enrollment, the senior investigators (Y.S., S.K., A.G., T.K., Y.N., M.S., Y.N., M.I., M.T., N.S., and S.N.) performed on-site auditing to ensure proper registration of each eligible patient. To ensure the accuracy of clinical events, the WET-HF registry is supported by a central study committee that adjudicates mode of death. Initially, all deaths were reviewed by investigators and then categorized into those in need of adjudication or those whose mode of death could be defined clearly. Central committee members reviewed the abstracted record and adjudicated mode of death. In this study, we analyzed data from 1343 consecutive AHF patients who were registered in the WET-HF2 between April 2018 and October 2019. Before the launch of the WET-HF2 registry, the objective and detailed design are provided for the clinical trial registration with the University Hospital Medical Information Network (UMIN000032169). The study protocol was approved by the institutional review boards at each site, and the research was conducted in accordance with the principles of the Declaration of Helsinki. According to the Ethical Guidelines for Medical and Health Research Involving Human Subjects and Personal Information Protection Law in Japan, informed consent was obtained from each subject before the study began.

### Study endpoints and definitions of pertinent variables

The study’s primary endpoint was a trend in tolvaptan usage for AHF patients after the JCS/JHFS 2017 guideline for HF was published. Secondary endpoints included the in-hospital mortality and LOHS, changes in both serum creatinine and natriuretic peptide levels, and also a trend in the severity of HF status defined by a validated risk model. The GWTG-HF risk score is a particularly useful tool for predicting in-hospital mortality in AHF patients, and it has been validated in the Japanese AHF patients^23,24^. The GWTG-HF risk score was calculated based on the seven variables at the time of admission including race (black or nonblack race), age, systolic blood pressure, heart rate, blood urea nitrogen, serum sodium, and presence of chronic obstructive pulmonary disease with and ranged from 0 to 101, with a higher score reflecting higher risk of mortality^23^.

Data on demographics, medical history, laboratory and other tests (such as electrocardiogram and echocardiogram), medications, procedures, and clinical outcomes during hospitalization and after discharge were recorded. Of note, vital signs, laboratory tests, and medications were evaluated at the time of admission and discharge. The New York Heart Association functional class was also evaluated at both admission and discharge by individual cardiologists at each institution and reviewed by the investigators. Left ventricular ejection fraction on echocardiogram was assessed by the modified Simpson’s method during the index hospitalization after the stabilization of HF signs and symptoms.

### Statistical analysis

The results are presented as medians with IQRs for continuous variables and as counts and percentages for categorical variables. Statistical comparisons between the two groups were performed using the Mann–Whitney U test for continuous variables and the Pearson’s chi-squared test for categorical variables. An association between use of tolvaptan and the in-hospital mortality and LOHS were evaluated using the multivariable logistic regression model adjusted for the GWTG-HF risk score. The generalized linear model was used to assess an association of use of tolvaptan with an improvement rate of NP levels ([NP values at admission − NP values at discharge]/NP values at admission) with an adjustment for the GWTG-HF risk score. We also performed the analysis of covariance (ANCOVA) to assess the association between use of tolvaptan and a change in serum creatinine levels from admission to discharge, adjusted for age, sex, and serum creatinine levels at baseline.

A time-trend of tolvaptan usage in the acute setting was described. We also parallelly examined a trend in the severity of HF status based on the GWTG-HF risk score among the two groups. The trend analysis of categorical variables was conducted by using the Cochran-Armitage test, and the trend of continuous variables were assessed by using linear regression model. All probability values were 2-tailed, values of p < 0.05 were considered statistically significant. All statistical analyses were performed with SPSS version 26.0 (SPSS Inc., Chicago, IL) and RStudio software, version 3.2.3.

## Supplementary Information

Supplementary Tables.
