# Supplementary material for: Temporal trends in tolvaptan use after revision of national heart failure guidelines in Japan
Source: Sci Rep. 2021 Sep 29;11:19360. doi: 10.1038/s41598-021-98173-8 (PMC8481277; doi:10.1038/s41598-021-98173-8)
Supplement: Supplementary file 1 — Supplementary Tables. [file 41598_2021_98173_MOESM1_ESM.docx]

**Table S1. Laboratory findings and medication patterns at hospital discharge**

|  | Tolvaptan (-)  n = 1,014 | Tolvaptan (+)  n = 249 | P value |
| --- | --- | --- | --- |
| Laboratory findings at discharge |  |  |  |
| Hemoglobin, g/dl | 12.0 (10.5–13.8) | 11.0 (9.9–12.8) | <0.001 |
| Creatinine, mg/dl | 1.04 (0.80–1.38) | 1.17 (0.85–1.66) | <0.001 |
| BUN, mg/dl | 22.2 (17.1–31.0) | 28.0 (19.0–41.1) | <0.001 |
| Sodium, mEq/l | 139.0 (137.0–141.0) | 139.0 (136.0–141.0) | 0.07 |
| Albumin, mg/dl | 3.4 (3.1–3.7) | 3.3 (3.0–3.7) | 0.07 |
| BNP, pg/ml * | 250 (117–482) | 345 (172–622) | <0.001 |
| NT-proBNP, pg/ml * | 1772 (870–4300) | 2642 (1319–5740) | 0.08 |
| Medication at discharge |  |  |  |
| ACEI or ARB, n (%) | 586 (58) | 122 (49) | 0.01 |
| Beta blocker, n (%) | 720 (71) | 185 (74) | 0.30 |
| MRA, n (%) | 359 (35) | 106 (43) | 0.04 |
| Digoxin, n (%) | 40 (4) | 9 (4) | 0.81 |
| Loop diuretics, n (%) | 828 (82) | 224 (90) | 0.002 |
| Furosemide equivalent, mg ^$^ | 30 (30–60) | 30 (30–60) | 0.25 |
| Thiazide-type diuretics, n (%) | 41 (4) | 13 (5) | 0.41 |

BUN, blood urea nitrogen; BNP, B-type natriuretic peptide; NT-proBNP, N-terminal pro-B-type natriuretic peptide; ACEI; angiotensin-converting enzyme inhibitor; ARB; angiotensin receptor blocker; MRA, mineralocorticoid receptor antagonist.

* In the 637 patients, BNP levels were measured, in contrast, NT-proBNP levels were measured in 186 patients.

^$^ Furosemide 20 mg = Azosemide 30 mg = Torsemide 4 mg

**Table S2. Baseline characteristics according the early (within 3 or less days) and late use (after 4 days) of tolvaptan**

|  | Early group  (n = 132) | Late group  (n = 135) | P value |
| --- | --- | --- | --- |
| Age, years | 80 (72–85) | 79 (70–85) | 0.52 |
| Men, n (%) | 78 (59) | 83 (61) | 0.69 |
| Body mass index, kg/m^2^ | 23.9 (20.6–27.3) | 23.3 (20.7–25.9) | 0.32 |
| Systolic BP, mm Hg | 132 (114–159) | 136 (117–156) | 0.37 |
| Heart rate, bpm | 87 (72–106) | 89 (75–105) | 0.67 |
| Left ventricular ejection fraction, % | 47 (31–57) | 45 (31–59) | 0.72 |
| GWTG-HF risk score | 43 (37–49) | 41 (35–47) | 0.07 |
| Etiology, n (%) |  |  | 0.27 |
| DCM | 14 (11) | 8 (6) |  |
| ICM | 31 (23) | 35 (26) |  |
| Valvular | 29 (22) | 44 (33) |  |
| Others | 58 (44) | 48 (36) |  |
| Comorbidities, n (%) |  |  |  |
| History of HF hospitalization | 53 (40) | 49 (36) | 0.51 |
| Coronary artery disease | 9 (7) | 5 (4) | 0.25 |
| Atrial fibrillation | 58 (44) | 60 (44) | 0.93 |
| Hypertension | 84 (64) | 82 (61) | 0.68 |
| Diabetes mellitus | 51 (39) | 38 (28) | 0.07 |
| Dyslipidemia | 53 (40) | 50 (37) | 0.60 |
| Stroke | 23 (17) | 18 (13) | 0.35 |
| COPD | 3 (2) | 6 (5) | 0.32 |
| Dementia | 4 (3) | 9 (7) | 0.17 |
| Laboratory findings |  |  |  |
| Hemoglobin, g/dl | 11.5 (9.4–13.1) | 11.6 (10.0–13.4) | 0.56 |
| Creatinine, mg/dl | 1.39 (0.96–1.93) | 1.03 (0.83–1.62) | <0.001 |
| BUN, mg/dl | 28.0 (19.1–42.6) | 23.2 (18.0–39.1) | 0.10 |
| Sodium, mEq/l | 139.0 (135.0–141.0) | 139.0 (137.0–141.0) | <0.001 |
| Albumin, mg/dl | 3.6 (3.2–3.9) | 3.5 (3.1–3.8) | 0.07 |
| BNP, pg/ml * | 813 (475–1370) | 844 (529–1625) | 0.50 |
| NT-proBNP, pg/ml * | 6790 (3013–12776) | 4383 (2189–8400) | 0.04 |
| Medication before admission, n (%) |  |  |  |
| ACEI or ARB | 47 (36) | 58 (43) | 0.22 |
| Beta blocker | 71 (54) | 61 (45) | 0.16 |
| MRA | 39 (30) | 34 (25) | 0.42 |
| Digoxin | 4 (3) | 6 (4) | 0.54 |
| Loop diuretics | 84 (64) | 78 (58) | 0.32 |
| Furosemide equivalent, mg ^$^ | 20 (0–40) | 10 (0–30) | 0.42 |
| Thiazide-type diuretics | 8 (6) | 4 (3) | 0.22 |
| In-hospital treatment, n (%) |  |  |  |
| Loop diuretics, iv | 108 (82) | 117 (87) | 0.28 |
| Vasodilators, iv | 41 (31) | 52 (39) | 0.20 |
| Inotropes, iv | 18 (14) | 17 (13) | 0.80 |
| Non-invasive ventilation | 60 (45) | 66 (49) | 0.57 |
| Intubation | 5 (4) | 6 (4) | 0.79 |
| IABP | 2 (2) | 4 (3) | 0.43 |
| VA-ECMO / VAD | 0 (<1) | 1 (<1) | 0.32 |

BP, blood pressure; DCM, dilated cardiomyopathy; ICM, ischemic cardiomyopathy; HF, heart failure; COPD, chronic obstructive pulmonary disease; eGFR, estimated glomerular filtration rate; BUN, blood urea nitrogen; BNP, B-type natriuretic peptide; NT-proBNP, N-terminal pro-B-type natriuretic peptide; ACEI; angiotensin-converting enzyme inhibitor; ARB; angiotensin receptor blocker; MRA, mineralocorticoid receptor antagonist; IABP, intraaortic balloon pumping; VA-ECMO, veno-arterial extracorporeal membrane oxygenation; VAD, ventricular assist device.

* In the 164 patients, BNP levels were measured, in contrast, NT-proBNP levels were measured in 103 patients.

^$^ Furosemide 20 mg = Azosemide 30 mg = Torsemide 4 mg
